# Osteoblast-derived WISP-1 increases VCAM-1 expression and enhances prostate cancer metastasis by down-regulating miR-126

**DOI:** 10.18632/oncotarget.2280

**Published:** 2014-07-30

**Authors:** Huai-Ching Tai, An-Chen Chang, Hong-Jeng Yu, Chao-Yuan Huang, Yu-Chieh Tsai, Yu-Wei Lai, Hui-Lung Sun, Chih-Hsin Tang, Shih-Wei Wang

**Affiliations:** ^1^ Department of Urology, National Taiwan University Hospital, Taipei, Taiwan; ^2^ Graduate Institute of Basic Medical Science, China Medical University, Taichung, Taiwan; ^3^ Department of Oncology, National Taiwan University Hospital, Taipei, Taiwan; ^4^ Division of Urology, Taipei City Hospital Renai Branch, Taipei, Taiwan; ^5^ Department of Molecular Virology, Immunology and Mediccal Genetics, Ohio state University, Columbus, OH, USA; ^6^ Department of Pharmacology, School of Medicine, China Medical University, Taichung, Taiwan; ^7^ Department of Biotechnology, College of Health Science, Asia University, Taichung, Taiwan; ^8^ Department of Medicine, Mackay Medical College, New Taipei City, Taiwan

**Keywords:** WISP-1, Osteoblasts, Prostate cancer, miR-126, VCAM-1

## Abstract

Bone metastases of prostate cancer (PCa) may cause intractable pain. Wnt-induced secreted protein-1 (WISP-1) belongs to the CCN family (CTGF/CYR61/NOV) that plays a key role in bone formation. We found that osteoblast-conditioned medium (OBCM) stimulates migration and vascular cell adhesion molecule-1 (VCAM-1) expression in human PCa (PC3 and DU145) cells. Osteoblast transfection with WISP-1 shRNA reduced OBCM-mediated PCa migration and VCAM-1 expression. Stimulation of PCa with OBCM or WISP-1 elevated focal adhesion kinase (FAK) and p38 phosphorylation. Either FAK and p38 inhibitors or siRNA abolished osteoblast-derived WISP-1-induced migration and VCAM-1 expression. Osteoblast-derived WISP-1 inhibited miR-126 expression. Moreover, miR-216 mimic reversed the WISP-1-enhanced migration and VCAM-1 expression. This study suggests that osteoblast-derived WISP-1 promotes migration and VCAM-1 expression in human PCa cells by down-regulating miR-126 expression via αvβ1 integrin, FAK, and p38 signaling pathways. Thus, WISP-1 may be a new molecular therapeutic target in PCa bone metastasis.

## INTRODUCTION

Prostate cancer (PCa) is the most commonly diagnosed malignancy in the United States and other Western countries [1]. During early stages, surgery is the most frequent therapeutic intervention. In advanced stages, however, systemic intervention is required to inhibit tumor growth and prevent secondary metastases. Bone metastasis is a common complication associated with advanced PCa, often causing acute pain and bone fracture. Bone metastasis has prognostic value in PCa, since the extent of disease in the bone significantly affects survival [2-4]. Metastasis to the bone, involving the osteoblasts and osteoclasts, results in bone lesions [5]. Osteoblasts, the major cellular component of bones, play a key role in osteogenesis [6, 7]. In the tumor microenvironment, cancer cells yield soluble factors to stimulate osteoblast activation, proliferation, and maturation. On the other hand, they secrete bone matrix and growth factors, which promote malignancy and osteoblastic bone metastasis [8, 9]. Therefore, osteoblast-derived factors are crucial during bone metastasis.

Wnt1-induced secreted protein-1 (WISP-1) is a cysteine-rich protein belonging to the Cyr61, CTGF, Nov (CCN) family of matricellular proteins that have developmental functions and regulate bone formation [10, 11]. CCN family proteins are mostly secreted and associated with the extracellular matrix (ECM), which has been demonstrated to play an important role in tumor development, including tumor survival, proliferation, migration, and invasion [12-14]. WISP-1 is reportedly expressed in developing breast tumors in transgenic mice [15]. Mounting evidence also suggests that WISP-1 enhances tumorigenesis and metastasis in many types of cancer [12, 16, 17]. This indicates that WISP-1 plays a vital role in cancer development and metastasis.

Several cell adhesion molecules secreted by cancer cells are involved in metastasis; e.g., integrin, cadherin, immunoglobulin superfamily [18, 19]. Vascular cell adhesion molecule-1 (VCAM-1 or CD106), a member of the immunoglobulin superfamily, is a transmembrane glycoprotein that mediates adhesion of lymphocytes or monocytes to the vascular endothelium [20]. Aberrant expression of VCAM-1 in cancer cells has been documented in preclinical models as well as patient samples of gastric cancer [21] and renal cell carcinoma [22]. Likewise, VCAM-1 has been indicated to regulate tumor progression and bone metastasis in glioblastoma and breast cancer [23-25]. It is unknown whether VCAM-1 has any functional role in PCa metastasis to the bone.

Bone-derived growth factor and chemokines play central roles as trophic factors that attract breast, lung, and prostate cancer cells to bone tissue [26]. Understanding the mechanism of interactions between human PCa and osteoblasts is critical to devising therapies for PCa bone metastasis. We hypothesized that osteoblast-derived WISP-1 may regulate VCAM-1 expression and promote the migration of PCa cells. We found that osteoblast-derived WISP-1 promoted PCa migration and VCAM-1 expression by down-regulating miR-126 through the αvβ1 integrin/focal adhesion kinase (FAK)/p38 signaling pathway.

## RESULTS

### Osteoblast-derived WISP-1 promotes prostate cancer cell motility

**Fig.1:**
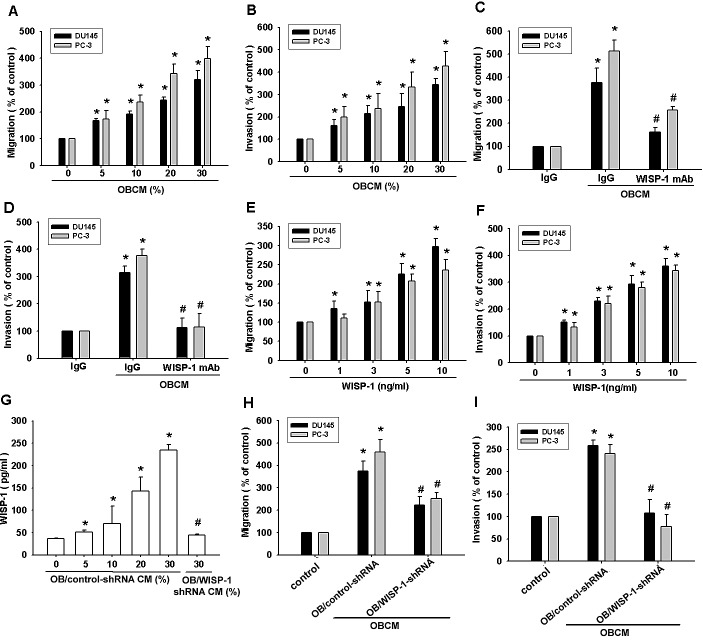
Osteoblast-derived WISP-1 increases prostate cancer motility (A–D) Prostate cancer (PCa) cells (DU145 and PC-3) were incubated with various concentrations of osteoblast conditional medium (OBCM) or OBCM pretreated with IgG or WISP-1 monoclonal antibody (10 μg/mL) for 30 min; *in vitro* migration and invasion were measured by a Transwell assay. (E, F) PCa cells were incubated with WISP-1 (1–10 ng/mL) for 24 h; *in vitro* migration and invasion were measured by a Transwell assay. (G–I) Osteoblasts were transfected with control or WISP-1 shRNA for 24 h, the medium was collected as OBCM, and WISP-1 expression was examined by ELISA. OBCM was applied to PCa cells for 24 h, and *in vitro* migration and invasion were measured by a Transwell assay. Results expressed as mean ± SEM. *, *p* < 0.05 compared with control; #, *p* < 0.05 compared with OBCM or WISP-1-treated group.

Bone-derived growth factors and chemokines reportedly promote tumor metastasis to the bone [7, 8, 28]. We hypothesized that osteoblasts are capable of regulating PCa metastasis to the bone and we examined the effect of OBCM on PCa cell motility via a Transwell assay. We found that migration and invasion of PCa cells (DU145 and PC-3) was OBCM concentration-dependent (Fig. 1A–B). It is well established that osteoblasts can synthesize and secrete WISP-1, which plays an important role in bone formation and cell differentiation [11, 29]. To pinpoint the contribution of WISP-1 in OBCM to PCa motility, we used a monoclonal antibody (mAb) against WISP-1. Figure 1C–D shows that WISP-1 mAb blocked OBCM-induced migration and invasion in PCa cells. To corroborate this hypothesis, we stimulated PCa cells with WISP-1 and found that this significantly increased migration and invasion in PCa cells (Fig. 1E–F). To confirm the role of WISP-1 in OBCM, we transfected osteoblasts with control or WISP-1 shRNA, collecting and applying their OBCM to PCa cells. As shown in Fig. 1G, transfection of osteoblasts with WISP-1 shRNA reduced WISP-1 expression in OBCM. WISP-1 shRNA also antagonized OBCM-mediated PCa migration and invasion (Fig. 1H–I). These results indicate that osteoblast-derived WISP-1 promotes migration and invasion in PCa cells.

### Osteoblast-derived WISP-1-directing prostate cancer migration involves VCAM-1 up-regulation through integrin αvβ1 receptor

**Fig.2:**
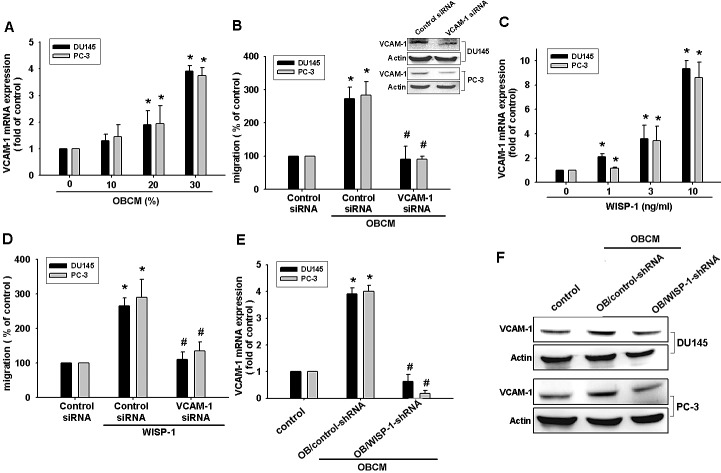
Vascular cell adhesion molecule-1 (VCAM-1) is involved in osteoblast-derived WISP-1-mediated PCa cell migration (A, C) PCa cells were incubated with various OBCM or WISP-1 concentrations for 24 h, and *VCAM-1* expression was examined by real-time quantitative polymerase chain reaction (RT-qPCR). (B, D) PCa cells were transfected with VCAM-1 siRNA for 24 h followed by stimulation with OBCM (30 %) or WISP-1 (10 ng/mL) for 24 h; *in vitro* migration was measured by a Transwell assay. (E, F) Osteoblasts were transfected with control or WISP-1 shRNA for 24 h, and the medium was collected as OBCM and applied to PCa cells for 24 h. VCAM-1 expression was examined by RT-qPCR and Western blot. Results are expressed as mean ± SEM. *, *p* < 0.05 compared with control; #, *p* < 0.05 compared with OBCM or WISP-1-treated group.

**Fig.3:**
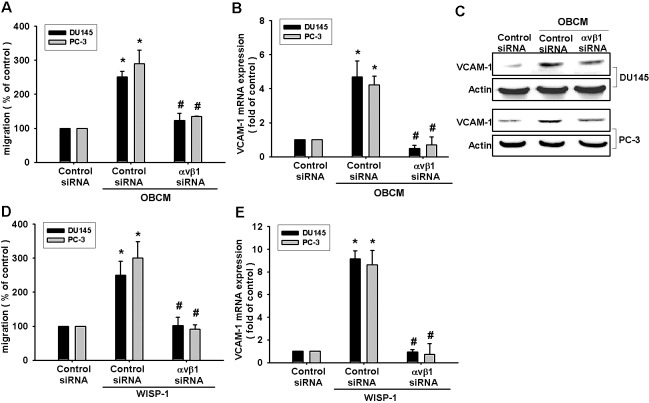
Osteoblast-derived WISP-1 boosts migration and VCAM-1 expression via integrin αvβ1 receptor (A–E) PCa cells were transfected with αvβ1 siRNA for 24 h followed by stimulation with OBCM (30 %) or WISP-1 (10 ng/mL) for 24 h; *in vitro* migration and VCAM-1 expression were determined by Transwell, RT-qPCR, and Western blot analyses. Results are expressed as mean ± SEM. *, *p* < 0.05 compared with control; #, *p* < 0.05 compared with OBCM or WISP-1-treated group.

VCAM-1 reportedly mediates tumor bone metastasis [25]. We hypothesized that VCAM-1 is involved in osteoblast-derived WISP-1-directed PCa migration. Stimulation of PCa cells with OBCM or WISP-1 increased *VCAM-1* mRNA expression in a concentration-dependent manner (Fig. 2A and C). Transfection of PCa cells with VCAM-1 siRNA markedly inhibited OBCM- or WISP-1-induced migration (Fig. 2B and D). WISP-1 shRNA antagonized OBCM-mediated VCAM-1 expression (Fig. 2E–F). These data suggest that OBCM-derived WISP-1-induced PCa migration occurs via up-regulation of VCAM-1 expression. WISP-1 is known to affect cell function by binding to the cell-surface integrin receptor [30]. Incubation of PCa cells with OBCM increased mRNA expression of αv and β1 integrin (data not shown). Co-transfection of PCa cells with αvβ1 siRNA markedly reduced OBCM- or WISP-1-enhanced cell migration (Fig. 3A and D). On the other hand, αvβ1 siRNA diminished OBCM- or WISP-1-mediated VCAM-1 expression (Fig. 3B, C, E). Thus, osteoblast-derived WISP-1 increases migration and VCAM-1 expression in human PCa cells through integrin αvβ1 receptor.

### FAK and p38 signal pathways are involved in osteoblast-derived WISP-1-mediated PCa migration and VCAM-1 expression

**Fig.4:**
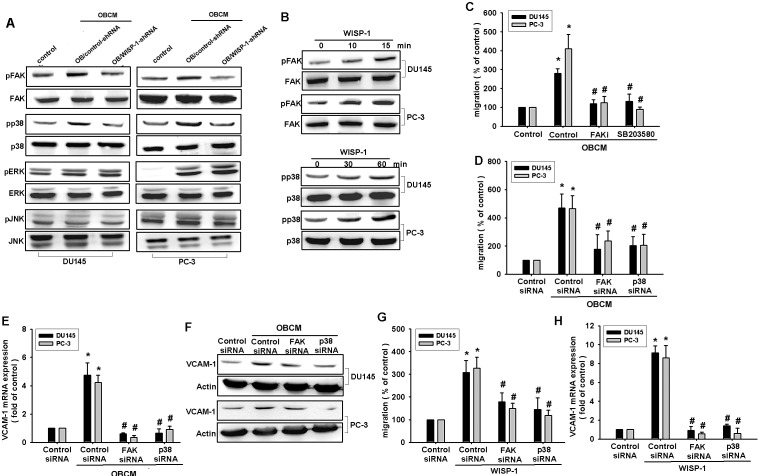
FAK and p38 pathways are involved in osteoblast-derived WISP-1-increased migration and VCAM-1 expression (A) Osteoblasts were transfected with control or WISP-1 shRNA for 24 h, and the medium was collected as OBCM and applied to PCa cells. FAK, p38, ERK, and JNK phosphorylation was examined by Western blot. (B) PCa cells were incubated with WISP-1 (10 ng/mL) for the indicated time intervals; FAK and p38 phosphorylation was examined by Western blot. (C–H) PCa cells were pretreated with FAK inhibitor (10 μM) and SB203580 (10 μM) or transfected with FAK and p38 siRNA for 24 h followed by stimulation with OBCM (30 %) or WISP-1 (10 ng/mL) for 24 h; *in vitro* migration and VCAM-1 expression were measured by Transwell, RT-qPCR, and Western blot analyses. Results are expressed as mean ± SEM. *, *p* < 0.05 compared with control; #, *p* < 0.05 compared with OBCM or WISP-1-treated group.

FAK, a widely expressed non-receptor protein tyrosine kinase, is an early downstream factor of integrin-mediated signaling that regulates cellular function [31]. To verify whether FAK activation is involved in osteoblast-derived WISP-1-induced cell migration, we directly measured phosphorylation of FAK in response to OBCM. Stimulation of PCa cells with OBCM increased FAK phosphorylation (Fig. 4A). In contrast, knockdown of WISP-1 in osteoblasts diminished OBCM-mediated FAK phosphorylation (Fig. 4A). FAK inhibitor or siRNA reduced OBCM- or WISP-1-increased cell migration and VCAM-1 expression in human PCa (Fig. 4C–H), indicating that WISP-1 enhanced FAK phosphorylation (Fig. 4B).

Mitogen-activated protein kinase (MAPK) activation is reported to be indispensible for migration in human PCa [32]. To delineate signal pathways downstream of WISP-1, we examined MAPK activity in OBCM-treated cells. Fig. 4A demonstrated that OBCM exposure caused an increase in the phosphorylation of ERK, p38, and JNK. Knockdown of WISP-1 reduced OBCM-mediated p38 but not ERK and JNK phosphorylation, indicating that p38 (not ERK or JNK) is involved in osteoblast-derived WISP-1-mediated cell function. Recombinant human WISP-1 promoted p38 phosphorylation in a time-dependent manner (Fig. 4B). To test the involvement of p38 in osteoblast-derived WISP-1-mediated migration, we used p38 inhibitor (SB203580) and siRNA. We found that p38 inhibitor or siRNA reduced OBCM- or WISP-1-induced cell migration and VCAM-1 expression (Fig. 4C–H), suggesting that the FAK and p38 pathways are involved in osteoblast-derived WISP-1-mediated migration and VCAM-1 expression in human PCa.

### Osteoblast-derived WISP-1 increased cell migration and VCAM-1 expression by down-regulating miR-126 through αvβ1 integrin, FAK, and p38 signaling pathways

**Fig.5:**
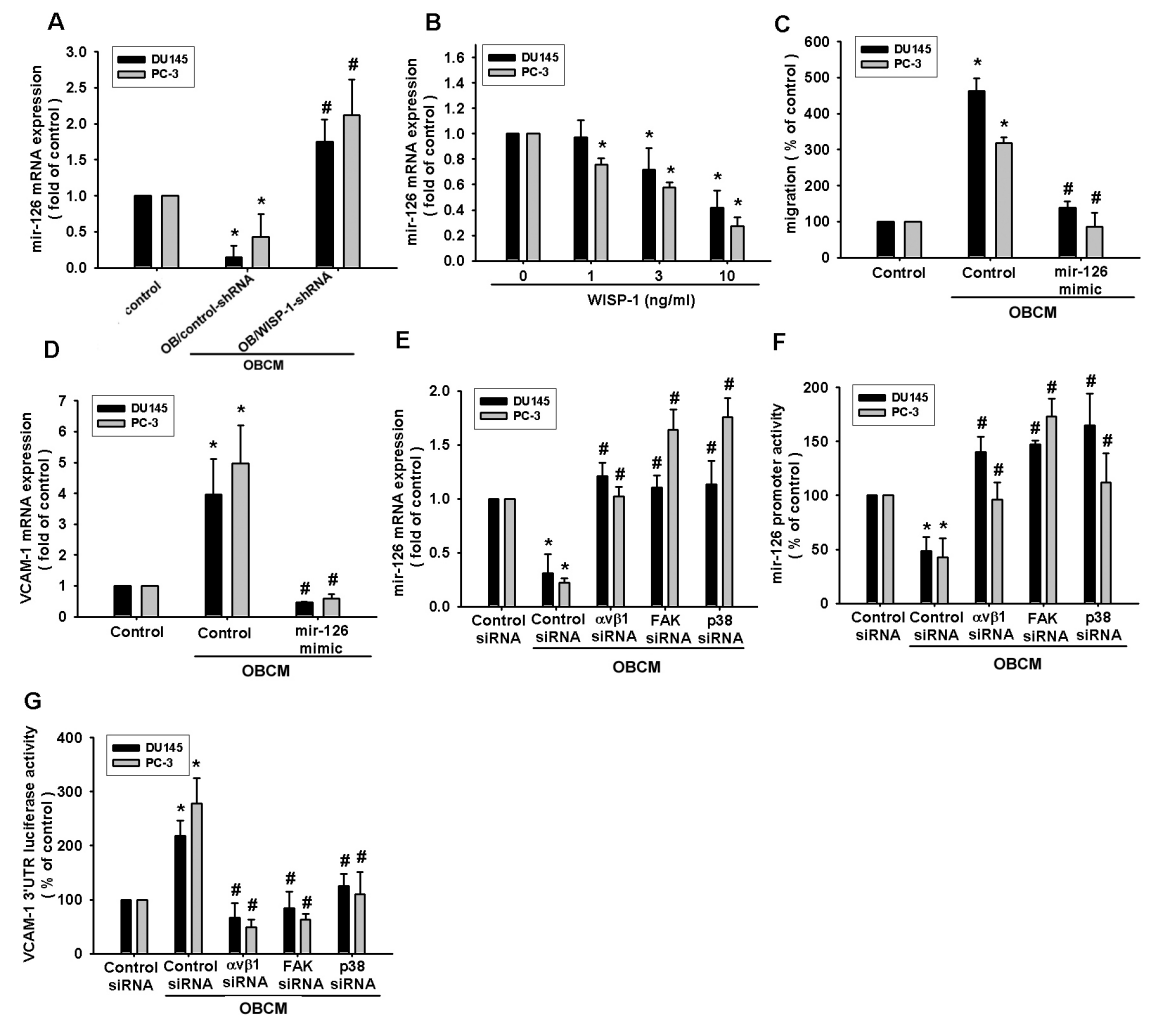
Osteoblast-derived WISP-1 boosts PCa migration and VCAM-1 expression by down-regulating miR-126 expression (A) Osteoblasts were transfected with control or WISP-1 shRNA for 24 h. Medium was collected as OBCM and applied to PCa cells; miR-126 expression was examined by qPCR. (B) PCa cells were incubated with WISP-1 (10 ng/mL) for the indicated time intervals, and miR-126 expression was examined by qPCR. (C, D) PCa cells were transfected with an miR-126 mimic for 24 h followed by stimulation with OBCM (30 %) or WISP-1 (10 ng/mL) for 24 h; miR-126 expression was examined by qPCR. (E–G) PCa cells were transfected with αvβ1, FAK, or p38 siRNA for 24 h followed by stimulation with OBCM (30 %) or WISP-1 (10 ng/mL) for 24 h, and miR-126 expression, miR-126 promoter activity, or *VCAM-1* 3′UTR activity were examined. Results are expressed as mean ± SEM. *, *p* < 0.05 compared with control; #, *p* < 0.05 compared with OBCM or WISP-1-treated group.

It has been reported that miR-126, regulating VCAM-1 expression, is involved in many cellular functions [33-35]. We hypothesized that miR-126, regulating VCAM-1, mediates osteoblast-derived WISP-1-induced cell migration. We found that incubation of PCa cells with OBCM reduced miR-126 expression, while WISP-1 shRNA rescued OBCM-inhibited miR-126 expression (Fig. 5A). Stimulation of PCa cells with WISP-1 inhibited miR-126 expression in a concentration-dependent manner (Fig. 5B). To confirm that miR-126 is involved in osteoblast-derived WISP-1-mediated cell migration, we used an miR-126 mimic and found that transfection with the miR-126 mimic inhibited OBCM-induced migration and VCAM-1 expression (Fig. 5C–D). Simultaneously, αvβ1, FAK, and p38 siRNA reversed OBCM-inhibited miR-126 expression and promoter activity (Fig. 5E–F), indicating that osteoblast-derived WISP-1 suppresses miR-216 via the αvβ1/FAK/p38 pathway. To examine whether miR-216 regulates the 3′UTR of *VCAM-1*, we constructed a luciferase-reporter vector harboring the 3′UTR of *VCAM-1* mRNA and another vector containing the miR-216-binding site. The data showed that OBCM increased luciferase activity in the *VCAM-1* 3′UTR plasmid, whereas αvβ1, FAK, or p38 siRNA reduced OBCM-mediated *VCAM-1* 3′UTR activity (Fig. 5G). Taken together, these data demonstrate that miR-216 directly represses VCAM-1 protein expression via binding to the 3′UTR of human *VCAM-1* through the αvβ1/FAK/p38 signaling pathway.

**Fig.6:**
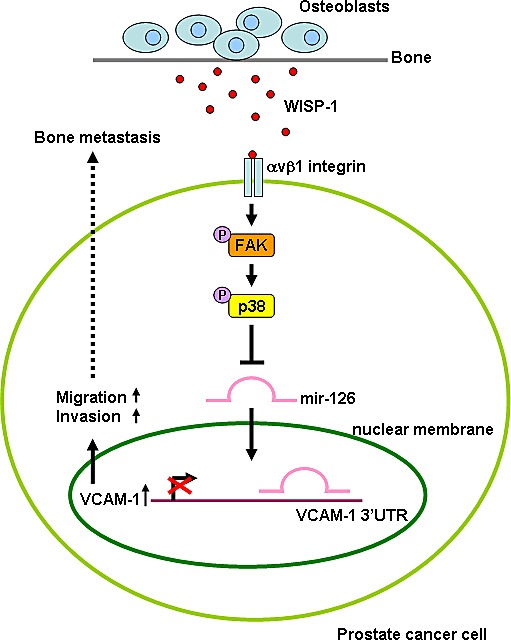
Schematic presentation of signaling pathways involved in osteoblast-derived WISP-1-induced migration and VCAM-1 expression of PCa cells Osteoblast-derived WISP-1 promotes migration and VCAM-1 expression in human PCa cells by down-regulating miR-126 expression via αvβ1 integrin, FAK, and p38 signaling pathways.

## DISCUSSION

PCa cells exhibit a remarkable tendency to metastasize to the bone [36-39]. Analysis of trophic signals that control PCa bone metastasis is crucial to identify new molecular targets for anti-metastasis therapy. We hypothesized that osteoblast-derived factors help to direct migration of PCa cells and found that osteoblast-derived factors induced migration of human PCa cells. Using WISP-1 shRNA to knock down WISP-1 expression in osteoblasts, we identified WISP-1 as the chief factor in osteoblasts that promotes PCa migration and VCAM-1 up-regulation. One mechanism we found that underlies osteoblast-derived WISP-1-directed migration was transcriptional up-regulation of VCAM-1 expression by down-regulation of miR-126 through αvβ1 integrin, FAK, and p38 signaling pathways.

Tumor invasion and metastasis are the main biological characteristics of cancer. Metastasis is the major cause of cancer-related death, involving multiple processes: invasion of cells and altering cell-cell adhesion properties, rearrangement of the ECM environment, suppression of anoikis, and reorganization of the cell cytoskeleton [40]. Cell adhesion molecules are transmembrane glycoproteins that mediate cell-cell and cell-ECM interactions. VCAM-1, a cell adhesion molecule, reportedly mediates epithelial-to-mesenchymal transition, invasion, and bone metastasis [23, 24]. In breast cancer, VCAM-1 is a crucial activator of indolent bone metastasis and osteoclast recruitment to form bone lesions. Pretreatment with VCAM-1 antibody reduces breast cancer cell migration and bone metastasis [25], indicating that VCAM-1 plays a critical role in tumor migration; its disruption can prevent bone metastasis. Our study uncovered evidence of VCAM-1 as a major factor in osteoblast-derived WISP-1-mediated migration in human PCa cells. We found that osteoblast-derived WISP-1 induced PCa cells to express VCAM-1, whereas siRNA against VCAM-1 significantly reduced WISP-1-mediated cell motility. VCAM-1 is thus a downstream effector in the osteoblast-derived WISP-1-increased motility of human PCa cells.

Newly identified small noncoding RNAs, miRNAs, belong to a novel class of gene regulators that control gene expression by binding to complementary sequences in the 3′UTRs of target mRNAs [41, 42]. Dysregulated miRNA expression has been reported in human cancer and may affect multiple steps during metastasis [43]. miR-126 has been reported as a negative regulator of VCAM-1 that mediates several cellular functions [33-35]. Our study defines a mechanism for miR-126 function wherein miR-126 mediates PCa migration by suppressing VCAM-1 expression. Our data show that osteoblast-derived WISP-1 inhibited miR-126 expression and promoter activity. Transfection with an miR-126 mimic halted osteoblast-derived WISP-1-mediated migration and VCAM-1 expression. Moreover, we showed that miR-126 directly repressed VCAM-1 protein expression through binding to the 3′-UTR of human *VCAM-1*, thereby negatively regulating VCAM-1-mediated metastasis.

Previous studies have shown that MAPK is activated after stimulation of CCN family proteins [26, 44]. We found that OBCM promoted ERK, p38, and JNK phosphorylation. However, knockdown of WISP-1 in osteoblasts decreased OBCM-induced p38 phosphorylation, while other MAPKs (JNK and ERK) were unaffected. This suggests that p38, but not JNK and ERK, is involved in osteoblast-derived WISP-1-mediated cell functions. In addition, p38 inhibitor reduced osteoblast-derived WISP-1-enhanced migration and VCAM-1 expression, as confirmed by inhibition of osteoblast-derived WISP-1-enhanced migration and VCAM-1 expression in human PCa by p38 siRNA. Transfection with αvβ1, FAK, or p38 siRNA diminished OBCM-mediated miR-126 expression and *VCAM-1* 3′UTR activity. Thus, our data indicate that αvβ1 integrin, FAK, and p38 signaling pathways are involved in osteoblast-derived WISP-1-inhibited miR-126 expression in human PCa cells.

Bone is a common site of cancer metastasis. PCa shows a particular predilection for metastasis to the bone. Bone-derived growth factors and chemokines play central roles as trophic factors that attract PCa cells to bone tissue [3]. Osteoblast-derived factors constitutively secreted by human osteoblasts play a key role of hematopoietic cells in the marrow [15]. The effect of osteoblast-derived factors on VCAM-1 expression and migration activity in human PCa cells remains mostly unknown. We observed that osteoblast-derived WISP-1 promoted migration and VCAM-1 expression in human PCa cells by down-regulating miR-126 expression via αvβ1 integrin, FAK, and p38 signaling pathways. Inhibition of WISP-1 thus presents a new, plausible therapeutic target in prostate cancer-related bone metastasis.

## MATERIALS and METHODS

### Materials

Anti-mouse and anti-rabbit IgG-conjugated horseradish peroxidase; rabbit polyclonal antibodies specific to VCAM-1, p-ERK, ERK, p-p38, p38, p-JNK, JNK, p-FAK, FAK, and β-actin; and WISP-1 short hairpin RNA (shRNA) and control shRNA plasmids were purchased from Santa Cruz Biotechnology (Santa Cruz, CA); p38 inhibitor (SB203580) was from Enzo Life Sciences (Farmingdale, NY); FAK inhibitor was from Calbiochem (San Diego, CA); recombinant human WISP-1 was from R&D Systems (Minneapolis, MN); Dulbecco's modified Eagle's medium (DMEM), RPMI-1640 medium, fetal bovine serum (FBS), and all other culture reagents were obtained from Gibco-BRL Life Technologies (Grand Island, NY); the luciferase assay kit was from Promega (Madison, WI); and miR-126 mimic and other chemicals were purchased from Sigma-Aldrich (St. Louis, MO).

### Cell culture

The human prostate cancer cell lines (PC3 and DU145) were purchased from American Type Culture Collection (Manassas, VA). Human primary osteoblasts were obtained from Lonza (Walkersville, MD). Cells were maintained at 37°C in a 5% CO2 atmosphere in RPMI-1640 medium supplemented with 20 mM HEPES, 10% heat-inactivated FBS, 2 mM glutamine, 100 U/mL penicillin, and 100 μg/mL streptomycin (Invitrogen; Carlsbad, CA). To obtain osteoblast-conditioned medium (OBCM), cells were grown to confluence and culture media were changed to RPMI without FBS. OBCM was collected two days after the medium change and stored at −70°C until use. In serial experiments, osteoblasts were transfected for 24 h with WISP-1 or control shRNA to prevent WISP-1 production and the medium was collected 48 h later. The level of WISP-1 in the culture medium was assayed with a WISP-1 enzyme-linked immunosorbent assay (ELISA) kit (R&D Systems; Minneapolis, MN), as per the manufacturer's instructions.

### Migration and invasion assay

Transwell inserts (Costar; New York, NY; pore size, 8 μm) in 24-well dishes were used for the migration assay. For invasion assays, filters were pre-coated with 30 μL Matrigel basement membrane matrix (BD Biosciences; Bedford, MA) for 30 min. Procedures were similar for the migration and invasion assays. Before the migration assay, cells were pretreated for 30 min with inhibitors (FAK inhibitor, SB203580, or vehicle control [0.1% dimethyl sulfoxide]), and 200 μL serum-free medium containing approximately 1×10^4^ cells was placed in the upper chamber, while 300 μL serum-free medium containing WISP-1 or OBCM was added to the lower chamber. Plates were incubated for 16 h at 37°C in 5% CO2, and cells were fixed in 3.7% formaldehyde solution for 15 min and stained with 0.05% crystal violet in phosphate-buffered saline (PBS) for 30 min. Cells on the upper side of filters were removed with cotton-tipped swabs and the filters were washed with PBS. Cells on the underside were examined and counted under a microscope. Each clone was plated in triplicate for each experiment, which was repeated at least three times.

### Quantitative real-time polymerase chain reaction (PCR)

Total RNA was extracted from cells using the TRIzol kit (MDBio Inc.; Taipei, Taiwan). Reverse transcription reactions were performed, in which 2 μg total RNA was reverse transcribed into cDNA using oligo (dT) primer. Quantitative real-time PCR (RT-qPCR) was conducted with the TaqMan^®^ one-step PCR Master Mix (Applied Biosystems; Foster City, CA). Total complementary DNA (100 ng/25-μL reaction) was mixed with sequence-specific primers and TaqMan^®^ probes, as per the manufacturer's instructions; sequences for all target gene primers and probes were purchased commercially (Applied Biosystems), and β-actin served as an internal control. q-PCR assays were carried out in triplicate, using a StepOnePlus sequence detection system. Cycling conditions were 10 min of polymerase activation at 95°C, followed by 40 cycles at 95°C for 15 s and 60°C for 60 s. Mir-X™ miRNA First-Strand Synthesis and SYBR^®^ RT-qPCR (Clontech Laboratories, Inc.; Mountain View, CA) kits were used for microRNA (miRNA) detection and reverse transcription, respectively. U6 snRNA levels served for normalization. A specific forward primer 5′-TCGTACCGTGAGTAATAATGCG-3′ was used for miR-126. Forward and reverse primers for U6 were 5′-CTCGCTTCGGCAGCACATATACTA-3′ and 5′-ACGAATTTGCGTGTCATCCTTGCG-3′. The threshold was set above the non-template control background and within the linear phase of target gene amplification to calculate the cycle number at which the transcript was detected (denoted as CT).

### Western blot analysis

Cellular lysates were prepared as described previously [27] while proteins were resolved by sodium dodecyl sulfate-polyacrylamide gel electrophoresis and transferred onto Immobilon polyvinyldifluoride membranes. Blots were blocked with 4% bovine serum albumin for 1 h at room temperature and probed with rabbit anti-human antibodies against β-actin, VCAM-1, p-p38, p38, p-JNK, JNK, p-FAK, FAK, p-ERK, or ERK (1:3000) for 2 h at room temperature. After three washings, blots were incubated with a donkey anti-rabbit peroxidase-conjugated secondary antibody (1:5000) for 1 h at room temperature. Blots were visualized using enhanced chemiluminescence and Kodak X-OMAT LS film (Eastman Kodak; Rochester, NY).

### Small interfering RNA (siRNA) transfection

The siRNAs against human FAK, p38, VCAM-1, and control siRNA were purchased from Santa Cruz Biotechnology. Cells were grown to 80% confluence in 6-well plates and then transfected with siRNAs (100 nM) by Lipofectamine 2000 Transfection Reagent (Invitrogen; Carlsbad, CA) for 24 h.

### Reporter gene assay

To construct miR-126 promoter-luciferase and *VCAM-1* 3′untranslated region (UTR)-luciferase plasmid, the 1.7-kb miR-126 promoter and *VCAM-1* 3′UTR fragments containing the miR-126-binding site GTATAGTACTGGCATGGTACGG were inserted into the multiple cloning site of pGL2-Basic vector containing a luciferase reporter gene. All constructs were sequenced and verified. Cells grown to 80% confluence in 12-well plates were transfected with 1 μg luciferase plasmid using Lipofectamine 2000. To prepare lysates, 100 μL reporter lysis buffer (Promega) was added to each well and cells were scraped from the dishes. The supernatant was collected after centrifugation at 13,000 rpm for 2 min. Aliquots of cell lysates (20 μL) containing equal amounts of protein (20–30 μg) were placed in wells of an opaque, black, 96-well plate; 80 μL luciferase substrate was added to all samples, and luminescence was measured in a microplate luminometer.

### Statistics

Data are presented as mean ± standard error of mean (SEM). The Student's t test was used for statistical analysis between samples. Comparison of more than two groups was performed using one-way ANOVA with Bonferroni's post-hoc test, where *p* < 0.05 was considered significant.
